# From Ovarian Hyperstimulation to the Discovery of a Liver Nodule

**DOI:** 10.7759/cureus.65381

**Published:** 2024-07-25

**Authors:** Mariana O Santos, Inês Marques, Carlos Barata, Maria Céu Almeida

**Affiliations:** 1 Obstetrics and Gynecology, Unidade Local de Saúde (ULS) de Coimbra, Coimbra, PRT; 2 Obstetrics, Maternidade Bissaya Barreto, Centro Hospitalar e Universitário de Coimbra, Coimbra, PRT

**Keywords:** liver nodules, pregnancy, thrombophilia, thrombosis, ovarian hyperstimulation

## Abstract

Ovarian hyperstimulation syndrome (OHSS) is a complication of ovulation induction. Deep vein thrombosis (DVT) can occur as a consequence of this syndrome, but it is an infrequent event. The authors describe the case of a woman who became pregnant after ovulation induction and developed severe OHSS and, subsequently, DVT of the right brachiocephalic trunk, internal and external jugular veins, and right subclavian vein. Thrombophilia studies were positive, revealing the presence of four mutations. The pregnancy was bichorionic and biamniotic twins and, during the course of the pregnancy, she developed severe cholestasis. In the follow-up of this situation, she underwent abdominal ultrasound which revealed the presence of liver nodules. Three years after delivery, the patient remains anticoagulated and under surveillance of liver nodules by annual MRI.

## Introduction

Ovarian hyperstimulation syndrome (OHSS) is a complication of ovulation induction, with its most severe form being rare. Thromboembolic events are the potentially most serious complication of this syndrome, but they are infrequent. They are mostly venous but can also occur in the arteries. Jugular or subclavian vein thrombosis occurs in 0.08 to 0.11 [1]. Risk factors for jugular or subclavian vein thrombosis in pregnant women are ovulation stimulation, use of a central venous catheter, thrombophilia, and malignancy [1,2]. Hereditary thrombophilias are conditions that increase the risk of thromboembolic events. During pregnancy, the hypercoagulable and hypofibrinolytic state makes this risk even greater [3]. Deep vein thrombosis (DVT) and pulmonary embolism are the best-documented complications.

## Case presentation

**Figure 1:**
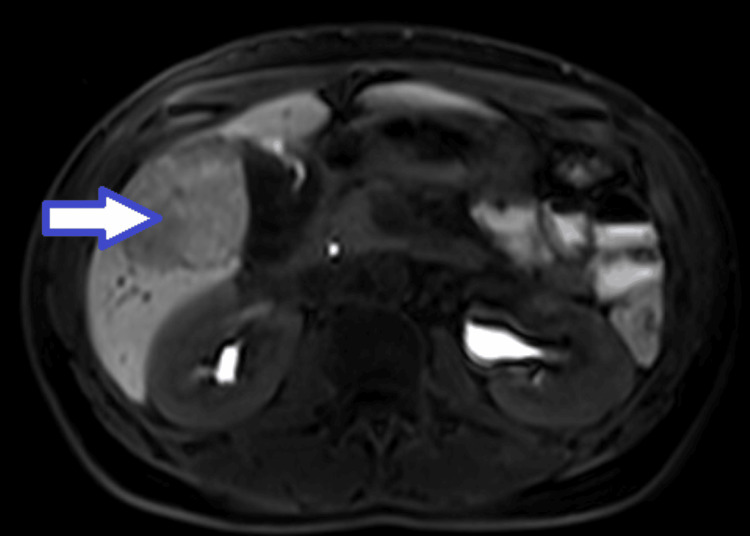
MRI image showing the hepatic nodule The arrow indicates the nodule

A 31-year-old patient was transferred from the hospital in her area of residence to Unidade Local de Saúde (ULS) de Coimbra due to severe OHSS, following ovulation induction with folitropin alfa 460 IU and corionadotrophin alfa 250 micrograms. On the day of admission, an ultrasound showed a uterus with a linear endometrium, bilaterally enlarged ovaries, and abundant pelvic effusion. Chest X-ray showed no abnormalities. In terms of laboratory tests, she had liver enzyme abnormalities (elevated transaminases), but the values were lower than those in the previous day's tests. She was medicated with enoxaparin 40 mg 2id, cabergoline, antiemetics, and analgesics. On the eighth day of hospitalization, two regular intrauterine gestational sac images were observed. She was discharged on the ninth day, with instructions to rest, analgesia, anticoagulation with enoxaparin 40 mg, protein, and fluid reinforcement. Two weeks after discharge from the Gynecology ward, she was hospitalized for three days in Vascular Surgery, due to evidence of DVT of the right brachiocephalic trunk, internal and external jugular veins, and right subclavian vein, and was medicated with enoxaparin 60 mg 2id. An ultrasound at nine weeks showed a developing bichorionic biamniotic twin pregnancy. She then continued her follow-up in the high-risk obstetrics appointment at Maternidade Bissaya Barreto. In the context of this consultation, she underwent thrombophilia studies which revealed the presence of double heterozygosity for Factor V Leiden and for PRT20210G/A and the presence of ANXA5 (M1/M2) and PAI-4G/4G. In a multidisciplinary meeting, it was decided to increase the enoxaparin dose to 70 mg twice a day, with control through the determination of the anti-Xa value. During the course of the pregnancy, she developed severe cholestasis and was medicated with ursodeoxycholic acid. In this context, she underwent abdominal ultrasound which revealed the presence of liver nodules. She was hospitalized at 31 weeks for threatened preterm labor and underwent two cycles of lung maturation and tocolysis. In August 2021, two live babies were born vaginally at 34 weeks: a boy weighing 1.585 g with an Apgar of 9/10/10 and a girl weighing 1.780 g with an Apgar of 9/10/10. After delivery, she underwent an MRI which revealed a "6.1 cm adenomatous lesion in segment V" (Figure 1).

**Figure 2:**
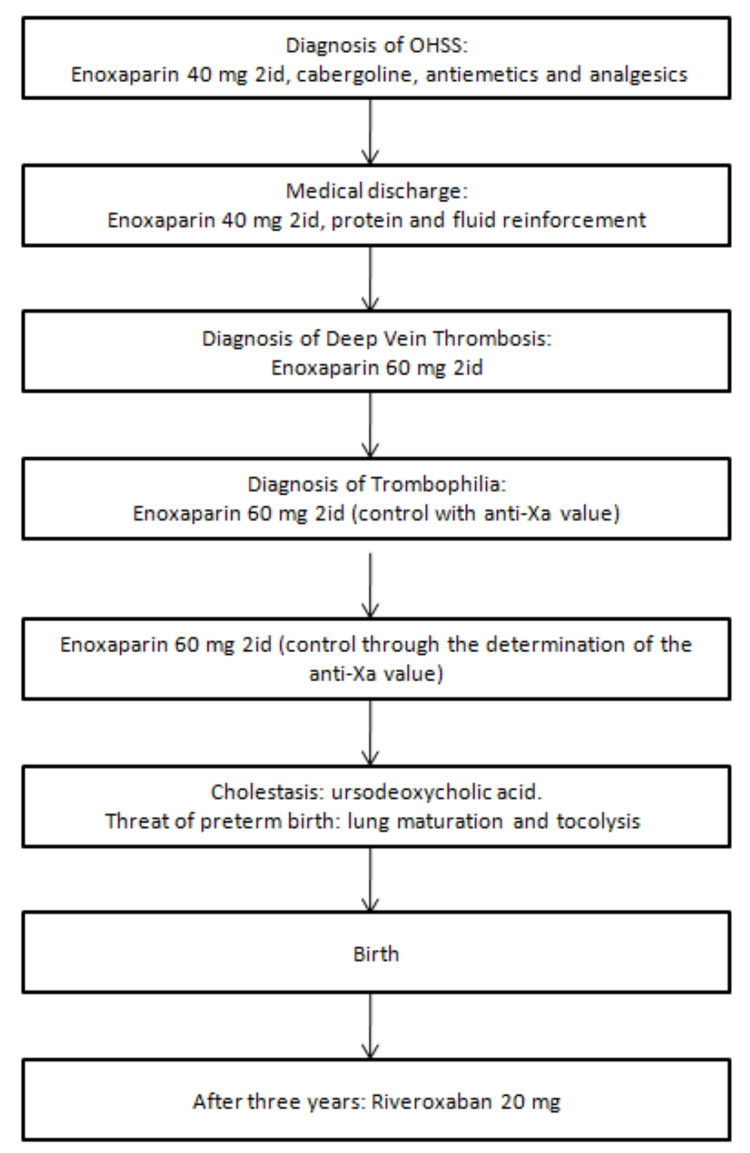
Timeline of clinical events OHSS: Ovarian hyperstimulation syndrome

The therapeutic decision was discussed in a meeting of the Hepato-Biliary Diseases Oncology Reference Center of ULS de Coimbra, and she was proposed for surgery due to suspicion of hepatocellular carcinoma. At her own discretion, she decided to seek a second opinion at another center and is still under annual surveillance with MRI for suspected adenoma or focal nodular hyperplasia. Currently, three years after delivery, the patient remains hypocoagulated with rivaroxaban 20 mg 2id (Figure 2) and is being followed up in hemostasis consultations at ULS de Coimbra.

## Discussion

OHSS is the most serious complication of ovulation induction. The administration of high doses of gonadotropin replaces the normal feedback mechanisms observed in the normal menstrual cycle, resulting in the formation of multiple follicles. There is then excessive production of VEGF, neovascularization, and increased capillary permeability. Fluid then shifts from the intravascular space to the third space, namely abdominal, pleural, and pericardial, in more severe cases. Hypovolemia and extravascular fluid lead to changes in organ function [4-6]. The frequency of this syndrome depends on the severity and the type of associated treatment, with the majority being mild or moderate. The severity of the syndrome is classified as mild, moderate, severe, and critical, depending on the clinical presentation, laboratory results, and imaging studies. The most severe forms include ascites, pleural effusion, oliguria/anuria, venous and arterial thrombosis, and sepsis. Mild cases require rest and analgesia. More severe cases require hospitalization. With regard to treatment, fluid replacement (intravenous in severe cases), analgesia, prophylactic anticoagulation in hospitalized women, and dopamine agonists (cabergoline 0.5 mg daily) are indicated. The resolution of the condition can occur on average in 15 days but may take longer in pregnant women. The enoxaparin dose should be adjusted based on anti-Xa activity determination, especially in cases where pharmacokinetics may be altered, such as pregnancy. The determination should be performed four hours after the last dose. This determination assesses the inhibition exerted by enoxaparin on factor Xa of the coagulation cascade. Hereditary thrombophilias are conditions that predispose an individual to a thrombotic event. Between 20 and 50% of pregnancies complicated by thrombosis have an identified thrombophilia. Factor V Leiden and prothrombin 20210G/A are the two most common. Factor V Leiden results from a mutation in the F5 gene that encodes factor V of the coagulation cascade and is the most common thrombophilia. Prothrombin is the precursor of thrombin, which is the final substance in the coagulation cascade and cleaves fibrinogen, giving rise to fibrin, which is deposited to form the fibrin clot. The mutation of the prothrombin gene results from the substitution of adenine for guanine at position 20210, with the majority being heterozygous. It is the second most common thrombophilia [7]. The presence of double heterozygosity for Factor V Leiden and PRT20210G/A is a cumulative risk factor for thrombotic events. With regard to thrombophilias, ANXA5 M1/M2 and PAI-4G/4G mutation are two risk factors associated with obstetric complications. ANXA5 is a protein with anticoagulant functions, expressed in large quantities in the syncytiotrophoblast. The presence of polymorphism in the PAI-1 gene can determine the presence of allelic variants (four or five guanine repeats-4G/5G), modifying gene expression. The 4G variant leads to a prothrombotic state due to increased expression of PAI-1 and decreased clot degradation [7,8]. 

## Conclusions

OHSS is a complication of ovulation induction. Thromboembolic events are the potentially most serious complication of this syndrome. Hereditary thrombophilias are conditions that increase the risk of thromboembolic events. During pregnancy, this risk is increased. Advances in imaging technology allow for the increasing detection of incidental liver lesions. Most lesions are cysts or hemangiomas and less frequently detected focal nodular hyperplasia or adenomas.
